# Editorial: mTOR pathway malfunctions in neurodevelopmental disorders

**DOI:** 10.3389/fnmol.2023.1131002

**Published:** 2023-01-17

**Authors:** Xinqi Fang, Hong Jiang, Xiaobing Zhang, Longbo Zhang

**Affiliations:** ^1^Department of Neurosurgery, Huashan Hospital, Fudan University, Shanghai, China; ^2^Department of Neurology, Xiangya Hospital, Central South University, Changsha, China; ^3^Department of Psychology, Florida State University, Tallahassee, FL, United States; ^4^Departments of Neurosurgery, and Cellular and Molecular Physiology, Yale School of Medicine, New Haven, CT, United States

**Keywords:** mTOR, neurodevelopmental disorders, epilepsy, microbiota, genomics

The Mechanistic Target of Rapamycin (mTOR) pathway integrates a variety of intracellular and extracellular signaling and coordinates many essential cellular processes, including protein synthesis and autophagy. During the neurodevelopment, mTOR acts as one of the main directors controlling the differentiation and proliferation of neural progenitor cells, neuronal morphology and migration, and synaptic plasticity (Costa-Mattioli and Monteggia, 2013). Malfunctions of mTOR signaling have been implicated across the continuum of neurodevelopmental and neuropsychiatric disorders (Nguyen and Bordey, 2021). mTORopathies such as tuberous sclerosis complex (TSC) and focal cortical dysplasia (FCD), are characterized by hyperactivation of mTOR signaling, malformation of cortical development, and disruptions of neural circuits and brain network leading to intractable epilepsy and cognition and behavior deficits (Crino, 2015). During last decades, extensive efforts on the mechanism studies have improved our understandings of the precise roles of mTOR involved in neurodevelopment and associated neurological disorders, and have promoted the development of targeted therapies.

In this Research Topic, a diverse insight into the different areas of research aimed at better understanding of neurological disorders caused by mTOR dysregulation. The original research by Riley et al. investigated the effects of mTOR on the development of inhibitory granule cells by generating a hyperactive mTOR complex 1 (mTORC1) model induced by homozygous *Tsc2* mutant. These results demonstrate that *Tsc2* has a critical role in regulating neural development and shapes inhibitory granule cells molecular and morphological characteristics. In addition, they performed a transcriptome profiling revealing a significantly different expression in the gene networks regulating neural circuitry following the loss of *Tsc2* gene. Ouyang et al. assessed GWAS data of epilepsy from the International League Against Epilepsy and gut microbiota from MiBioGen, and applied Mendelian Randomization analysis to explore the potential causal relationship among the gut microbiota, metabolites, and epilepsy. The study allows to explore the etiology of epilepsy from a new dimension. In the review articles, Girodengo et al. summarized the accumulating evidence from single-cell transcriptomics and cerebral organoid-based studies to improve our understanding of the crucial role of mTOR pathway in brain development and neurological disorders. Furthermore, they highlighted new ultra-sensitive techniques for the identification of somatic mTOR pathway mutations shedding light on the neurodevelopmental origin and phenotypic heterogeneity in mTORopathy patients. Wang et al. reviewed the current advances in imaging genetics in brain microcircuits and epileptic networks during brain development. The imaging genetics analysis accurately and efficiently integrates multidimensional datasets within a unified framework, providing a unique opportunity to generate a global vision for epilepsy to promote the molecular mechanism exploration and develop targeted treatment strategies.

In summary, the present Research Topic comprises original research and comprehensive reviews highlighting the role of mTOR signaling in neurodevelopment disorders. We hope that this topic will inspire further mechanism exploration of mTOR with the aim of fully restoring function to those neurological disorders.

## Author contributions

All authors listed have made a substantial, direct, and intellectual contribution to the work and approved it for publication.
